# Motivation rulers for smoking cessation: a prospective observational examination of construct and predictive validity

**DOI:** 10.1186/1940-0640-7-8

**Published:** 2012-06-08

**Authors:** Edwin D Boudreaux, Ashley Sullivan, Beau Abar, Steven L Bernstein, Adit A Ginde, Carlos A Camargo

**Affiliations:** 1 Departments of Emergency Medicine, Psychiatry, and Quantitative Health Sciences, University of Massachusetts Medical School, Worcester, MA, 01655, USA; 2 Department of Emergency Medicine, Massachusetts General Hospital, Boston, MA, 02114, USA; 3 Department of Emergency Medicine, Yale University School of Medicine, New Haven, CT, 06510, USA; 4 Department of Emergency Medicine, University of Colorado School of Medicine, Aurora, CO, 80045, USA

**Keywords:** Tobacco, Tobacco cessation, Motivation, Stage of change, Reliability, Validity

## Abstract

**Background:**

Although popular clinically, the psychometric properties of motivation rulers for tobacco cessation are unknown. This study examined the psychometric properties of rulers assessing importance, readiness, and confidence in tobacco cessation.

**Methods:**

This observational study of current smokers was conducted at 10 US emergency departments (EDs). Subjects were assessed during their ED visit (baseline) and reassessed two weeks later. We examined intercorrelations between the rulers as well as their construct and predictive validity. Hierarchical multinomial logistic regressions were used to examine the rulers’ predictive ability after controlling for covariables.

**Results:**

We enrolled 375 subjects. The correlations between the three rulers ranged from 0.50 (between Important and Confidence) to 0.70 (between Readiness and Confidence); all were significant (p < 0.001). Individuals in the preparation stage displayed the highest motivation-ruler ratings (all rulers F _2, 363_ ≥ 43; p < 0.001). After adjusting for covariables, each of the rulers significantly improved prediction of smoking behavior change. The strength of their predictive ability was on par with that of stage of change.

**Conclusion:**

Our results provide preliminary support for the psychometric soundness of the importance, readiness, and confidence rulers.

## Introduction

The construct of motivational readiness to change is fundamental to many health behavior theories, and it lies at the heart of numerous therapeutic approaches. For example, motivational interviewing [1] (MI) focuses on enhancing internal motivation to change a behavior and emphasizes that, when strong internal motivation is present, behavior change is more likely to occur and to persist. Motivational interviewing and other types of brief intervention (BI) derived from it have enjoyed considerable empirical support across a range of settings, study samples, and target behaviors [1,2] and have been successfully applied to smoking cessation [3-6].

The practical application of MI in clinical settings (as well as interventions derived from other health behavior theories such as the transtheoretical model [7]) typically includes the regular assessment of motivation. Motivational assessments can help clinicians tailor the therapeutic approach, exercises, homework, duration, and resources used with an individual. Although clinicians working in specialized outpatient settings to treat tobacco use can use more comprehensive measures of motivation, clinicians working in medical settings, such as primary care clinics, hospitals, and EDs, require very short and rapid assessment tools. Assessments that can be condensed to a few easy-to-understand questions and that can be re-administered over time while maintaining their reliability and validity are considered the most useful.

Rulers assessing importance, readiness, and confidence in quitting can rapidly assess these three dimensions of motivation. They are described by Miller and Rollnick [1,8] and have been incorporated into brief MI-based intervention protocols [3,9,10]. Motivation rulers have become popular clinically [11]. They have been widely disseminated, do not require scoring or the use of algorithms, take only a short time to complete, and are familiar to patients and providers alike due to the common use of similar kinds of scales to assess medical symptoms like pain. However, few published studies have examined their psychometrics, either in the broad field of substance abuse or in the subfield of tobacco cessation. Biener and Abrams [12] demonstrated that, while an 11-rung Contemplation Ladder was predictive of readiness to quit smoking and participation in educational programs about smoking, it did not predict biochemically validated abstinence. In contrast, a later study by Abrams and colleagues [13] did find that the Contemplation Ladder predicted smoking status at one- and two-year follow-up. Most recently, Chung and colleagues [14] studied a sample of 154 adolescents and found that 10-point rulers measuring readiness to quit as well as motivation, confidence, and difficulty in abstaining were all predictive of total number of cigarettes smoked during a 30-day timeline follow-back period as assessed 12 months after the initial ratings were collected. The existing literature, though small, suggests further study of the psychometrics of motivation rulers is important. In particular, studies are needed that replicate the validity of the rulers in different populations and that help to tease apart how the rulers relate to one another, how they compare to other indices of motivation, and whether they are able to predict not only readiness to change but actual behavior change as well.

To further explore the psychometrics of importance, readiness, and confidence rulers for tobacco cessation, and to advance our understanding of how motivational readiness relates to behavior change in general, we examined 1) the correlation across the three rulers to assess independence of the measures; 2) the correlations between the rulers and stages of change in the transtheoretical model (i.e., convergent validity); and 3) the ability of the rulers to predict smoking behavior in the two weeks following baseline assessment (i.e., predictive validity).

## Methods

### Procedures and participants

This study was part of a large prospective cohort study conducted in 2008–2009 using subjects recruited from 10 EDs in eight geographically diverse US states. The parent study was primarily interested in examining patterns of smoking behavior in the six months following an ED visit under treatment-as-usual conditions to help plan and power a future randomized controlled trial. In addition to estimating cessation-relapse patterns under naturalistic conditions, we intended to examine the association of a variety of baseline predictors with both short- and long-term change. This analysis focused on the associations between the motivation indicators and short-term change.

During a 10-day enrollment period, trained research staff screened consecutive ED patients for tobacco use. Patients were recruited during peak volume hours (9:00 AM to midnight). Each of the 10 sites enrolled a minimum of 36 subjects. Eligible subjects were 18 years or older and currently smoked cigarettes. Although participants had to have smoked >100 cigarettes in their lifetime (i.e., had to be an ever smoker), there was no minimum smoking rate, and we enrolled both nondaily and daily smokers. We excluded potential subjects with illnesses that precluded conversation or adequate comprehension of the study requirements, including those with altered mental status, acute intoxication, hostile or agitated behavior, an insurmountable language barrier, or severe illness (e.g., intubation, persistent vomiting). In addition, subjects at high risk of being lost to follow-up were excluded, including those who had transient residence and no access to a telephone. Sites maintained a registry that recorded all patients registered in the ED during the shift to facilitate a comparison of enrolled patients with those not enrolled.

Subjects completed a paper-and-pencil baseline self-report assessment in the ED, which provided data on smoking-related variables and predictors of cessation. All measures were printed in both English and Spanish. As an alternative, to accommodate patients who had poor eyesight or were illiterate, the assessment could be completed via interview with a research-staff member. To reduce demand bias, which could lead to under-reporting of tobacco use and over-reporting of interest in cessation, participants were reassured that their responses would not be shared with their treating clinicians.

All subjects received treatment as usual by their medical providers for their tobacco use. The research staff did not provide any counseling; however, after baseline data collection was complete, they did give subjects an educational pamphlet on smoking cessation published by the US Department of Health and Human Services and a list of tobacco-cessation treatment options, including the National Quitline number. Furthermore, subjects who screened positive for depression, alcohol, or drug use (screening was included as part of the full baseline assessment) were given the respective educational pamphlet published by the Association for Behavioral and Cognitive Therapies (ww.abct.org) as well as brochures with national mental-health hotlines and state-based behavioral health referral services.

Research staff at each site completed telephone follow-up interviews two weeks, three months, and six months after the index ED visit. A call window of seven days was used at each timepoint. Only the two week follow-up data was used in the current analysis, because we were interested in studying change immediately after the ED visit to guide the modeling of predictors for the three- and six-month analyses. Two weeks was chosen as the first assessment point because it allowed us to examine the range of behavior change from ongoing smoking, to a quit attempt with relapse, to transition to seven-day point prevalence abstinence within a short time period to maximize accuracy of recall.

The study was coordinated by the Emergency Medicine Network (EMNet). Data-collection forms were reviewed by EMNet staff, and missing or inconsistent data were reconciled through communication with the study site. All data underwent double data entry. The institutional review boards at all 10 sites approved the study. Participants provided written informed consent.

### Measures

The specific measures used for this paper represent a subset of the full battery.

#### Demographics

At the index ED visit (baseline), we collected data on age, sex, race/ethnicity, and educational level.

#### Nicotine dependence

At baseline, nicotine dependence was assessed using the Heavy Smoking Index [15], a well-established self-report measure of nicotine dependence for use when rapid assessment is needed. Strength of nicotine dependence is represented on the index by the sum of cigarettes smoked per day and the time until first cigarette. Scores between zero and three indicate low to moderate dependence, and scores greater than three indicate high dependence. The Heavy Smoking Index correlates highly with the Fagerström Test for Nicotine Dependence and is positively associated with carbon monoxide levels [15].

#### Stage of change

At baseline, stage of change [7] was assessed by asking subjects if they intended to quit smoking, and, if so, when they intended to do so given the following options: >12 months from now, within 6 to 12 months, within 1–6 months, within the next 30 days, or today. Stage of change was defined using this intention variable and the 24-hour quit attempt variable described above: precontemplation (no intention to quit within the next six months); contemplation (intention to quit within the next six months); and preparation (intention to quit within the next 30 days and a quit attempt within the past 12 months).

#### Motivation rulers

Participants rated three rulers associated with motivation to quit smoking at baseline. Importance was indexed by, “How important is stopping smoking to you (0 = Not important at all; 10 = Most important goal of my life)?” Readiness was indexed by, “How ready are you to quit smoking within the next month (0 = Not at all; 10 = 100% ready)?” Confidence was indexed by, “How confident are you that you will quit smoking within the next month (0 = Not at all; 10 = 100% confident)?” Although these rulers were patterned after recommendations by Miller and Rollnick [1,8]. they differ slightly. We used the lowest response option of “0” rather than “1.” We chose “0” because the value labels anchoring the low end of the scale referred to “not at all,” suggesting a complete absence of the ruler’s construct. We felt “0” to be a more accurate representation of this state than “1.” In addition, a 0–10 scale has the advantage of having a true midpoint represented by a whole number (“5”), versus the true midpoint of a 1–10 scale, which is “5.5.”

#### Follow-up assessment measures

Two weeks after the ED visit, patients were called by research staff to assess their smoking behavior, including whether they smoked daily, some days, or not at all. They were asked whether they had gone 24 hours without smoking *because they were trying to quit* (a quit attempt). In addition, subjects reported whether they had smoked, even a puff, in the past seven days (seven-day point prevalence abstinence—a common threshold set by clinical trials to consider someone a successful changer). Following previous ED-based studies [16], all subjects were categorized as 1) having no quit attempt >24 hours (continuous smokers); 2) having a quit attempt with relapse back to smoking by the two-week follow-up assessment (relapsers); or 3) reporting seven-day point prevalence abstinence at the two-week follow-up assessment (successful changers). The interview did not assess whether the individual had less than seven days of abstinence at follow-up; however, we did ask whether individuals currently smoked every day, some days, or not at all. A small number of participants (n = 2) reported no smoking but did not report seven-day point prevalence abstinence, suggesting that these individuals may have had fewer than seven days of abstinence. For the purposes of this study, these individuals were considered continuous smokers. All analyses were also completed with these two subjects removed, and none of the results changed substantively. We decided against including biochemical validation of self-reported smoking because misclassification rates among smokers are generally so low as to not materially affect conclusions from most nontreatment studies [17].

## Data analysis

The goal of the data analyses was to examine the relationship between the motivation rulers, stage of change, and behavior change after an ED visit. To examine the inter-relation between the three rulers (importance, readiness, and confidence), and thereby derive a measure of their independence, we calculated Pearson correlation coefficients. To examine convergent validity, we computed associations between the three rulers and stage of change using chi-square analyses. We hypothesized that there would be a positive association between the three rulers and stage of change. As exploratory analyses, we examined the rulers’ associations with other baseline variables, including age, sex, education level, race, and nicotine dependence.

To examine predictive validity, we first examined the bivariable relations between the rulers and smoking behavior change assessed at the two-week follow-up (continuous smokers, relapsers, and successful changers). We expected the rulers to predict smoking behavior change, with higher scores being associated with a greater likelihood of attempting to quit. Moreover, among those attempting to quit, we expected higher scores to be associated with transitioning to successful change (versus relapsing back to smoking).

We computed hierarchical multinomial logistic regressions to further evaluate the relative strength of the rulers to predict smoking behavior after adjusting for potential confounders. In the first model (examining the relationship between the Importance Ruler and smoking behavior), age, sex, race, educational level, and nicotine dependence were included in Step 1 as control variables, and the Importance Ruler was included at Step 2. We ran four more identical models but replaced the Importance Ruler with one of the other motivation variables: model 2 = Readiness Ruler, model 3 = Confidence Ruler, model 4 = all three rulers combined, and model 5 = Stage of Change. Separate models were run due to the high collinearity between the rulers and stage of change. This allowed a side-by-side comparison of the relative strength of each ruler and stage of change in predicting behavior change, after controlling for covariables. Data were analyzed using IBM® SPSS Statistics 19 (Armonk, NY).

## Results

### Descriptives

Of the 3662 consecutive patients screened for the study, 2132 (58%) were nonsmokers; 590 (16%) had a medical, psychological, or mental-status problem preventing approach; 192 (5%) refused to be screened; 92 (3%) had an insurmountable language barrier; 106 (3%) were unable or unwilling to be followed over time; and 172 (5%) were not enrolled for other miscellaneous reasons (e.g., left against medical advice, under state custody, discharged prior to approach). Although 378 patients enrolled into the study, three subjects were removed because of missing data, leaving 375 for analysis. The characteristics of study participants are shown in Table 1.

**Table 1:** Descriptive Characteristics of Predictor Variables (N = 375)

**Predictors**	**Frequencies (%)**	**Mean (SD)**
*Demographics*		
Age (in years)		41 (12)
Sex:		
Male	164 (44%)	
Female	210 (56%)	
Race:		
White	152 (41%)	
Nonwhite	220 (59%)	
Educational Status:		
<High School	88 (24%)	
High School Graduate	282 (75%)	
*Predictors*		
Nicotine Dependence		2.50 (1.62)
Importance Ruler^1^		7.31 (3.06)
Readiness Ruler^2^		6.16 (3.38)
Confidence Ruler^3^		4.63 (5.00)
Stage of Change:		
Precontemplation	166 (44%)	
Contemplation	122 (33%)	
Preparation	78 (21%)	
*Smoking Behavior Change (Two-Week Follow-Up)*		
Quit Attempt^4^	121 (32%)	
Seven-Day Point Prevalence Abstinence^5^	24 (6%)	
Smoking Behavior Change^6^:		
Continuous smoker	254 (68%)	
Relapse	97 (26%)	
Successful change	24 (6%)	

^1^Importance ruler: 0 = “not at all important”; 10 = “the most important goal of my life.” ^2^Readiness ruler: 0 = “not at all ready”; 10 = “100% ready.” ^3^Confidence ruler: 0 = “not at all confident”; 10 = “100 % confident.” ^4^Quit attempt: 0 = Did not stop smoking for one day because trying to quit; 1 = Stopped smoking for one day because trying to quit. ^5^Seven-day point prevalence abstinence: 0 = Smoked at least a puff in the last seven days; 1 = Did not smoke even a puff in the last seven days. ^6^Smoking behavior change: relapse = a 24-hour quit attempt but back to smoking at two-week follow-up; continuous smoker = no 24-hour quit attempt; successful change = seven-day point prevalence abstinence at two-week follow-up.

Compared with patients who were not enrolled (i.e., were not eligible, were not approached, or refused), those enrolled were more likely to be younger, to have Medicaid insurance, and to be discharged from the ED versus admitted (p < 0.05 for all; data not shown). No differences were observed between those enrolled and those not enrolled with regard to sex or race/ethnicity.

The average baseline Heavy Smoking Index score of the sample corresponded to low nicotine dependence. In terms of the motivation rulers, participants tended to report higher importance than readiness and higher readiness than confidence. A total of 149 participants (40%) reported that quitting smoking was “the most important goal of my life,” and 107 (29%) indicated they were “100% ready.” A much smaller proportion (n = 57; 15%) indicated they were “100% confident” in their ability to quit. Regarding motivation to quit, the largest proportion of participants (44%) fell in the precontemplation stage at baseline, followed by contemplation (33%) and preparation (21%).

### Relations among the rulers

The bivariable correlation between importance and readiness was 0.68 (p < 0.001), between readiness and confidence was 0.70 (p < 0.001), and between importance and confidence was 0.50 (p < 0.001).

### Ruler associations with stage of change

**Figure 1:**
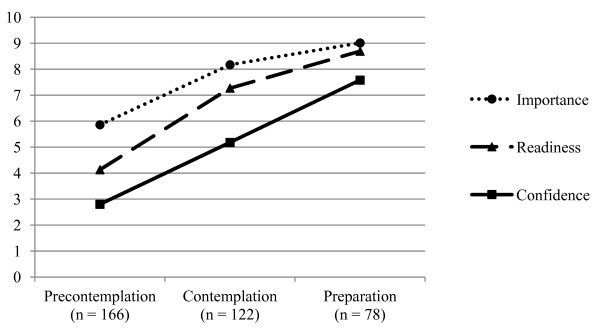
Stage of change and motivation rulers.

Individuals in the preparation stage displayed the highest level on each of the rulers, while individuals in the precontemplation stage displayed the lowest (all F _2, 363_ ≥ 42, all p < 0.001) (Figure 1).

### Ruler associations with other variables

Readiness was the only ruler associated with age, such that older participants were more ready to quit (r = 0.12, p < 0.05). There were no associations between the three individual motivation rulers and participant sex. Each ruler was associated with race and educational status, such that nonwhite participants (t _370_ = 4.60, 3.82, and 5.21, respectively; all p < 0.001) and individuals with less than a high school education (t _368_ = 3.00, 3.07, and 2.32, respectively; all p < 0.05) reported higher levels of importance, readiness, and confidence. Participants who reported stronger nicotine dependence reported lower levels of each of the rulers (r = −0.14, -0.22, and −0.28, respectively; all p < 0.01).

### Bivariable predictors of smoking behavior

A total of 244 participants were successfully contacted at the two-week follow-up, representing a 65% retention rate. We employed intention-to-treat principles in our analytic plan, such that all individuals lost to follow-up were assumed to have experienced the least desirable outcome, i.e., continuous smoking. We also performed a multiple imputation analysis pooling across five imputed datasets. The results from the two sets of analyses were substantively identical, so we discuss only the results from the more conservative intention-to-treat analyses.

Age and education were not significantly associated with smoking behavior change (all p > 0.10). Sex was related to smoking behavior change (χ² _2_ = 8.39, p < 0.05), such that women (n = 66; 31%) were more likely than men (n = 30; 18%) to have attempted to quit and relapsed. Race was predictive of smoking behavior change (χ² _2_ = 12.51, p < 0.01), such that nonwhite participants (n = 69; 31%) were more likely to attempt to quit and relapse than white participants (*n* = 26; 17%). Nicotine dependence also predicted smoking behavior change (F _2, 358_ = 5.38, p < 0.01), with the highest baseline dependence seen among continuous smokers and the lowest among successful changers.

**Figure 2:**
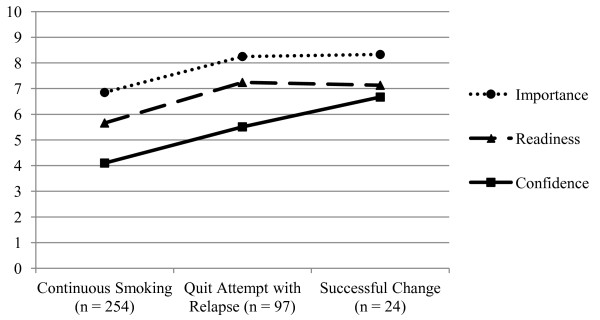
Smoking behavior change and motivation rulers.

Each ruler was associated with smoking behavior change (Importance—F _2, 372_ = 9.09; Readiness—F _2, 372_ = 9.01; Confidence—F _2, 372_ = 10.81; all p < 0.001) (Figure 2). Post-hoc tests revealed that those with a quit attempt (i.e., relapsers and successful-change) displayed higher confidence than continuous smokers (M _differences_ > 1.41; Tukey’s Honestly Significant Difference [HSD] test p values, < 0.01). Relapsers also displayed significantly greater importance and readiness than continuous smokers (M _differences_ > 1.40, Tukey’s HSD p values, < 0.01). Successful changers displayed higher mean levels of importance and readiness than continuous smokers (M _differences_ > 1.46) as well as higher confidence than relapsers (M _differences_ = 1.16), but these differences did not reach statistical significance (Tukey HSD p values, > 0.05).

Stage of change was strongly predictive of smoking behavior change (χ² _4_ = 40.41, p < 0.001), with individuals in the contemplation (n = 39; 32%) and preparation (n = 28; 36%) stages more likely to attempt and relapse than individuals in the precontemplation (n = 28; 17%) stage, and individuals in the preparation (n = 14; 18%) stage more likely to successfully change than individuals in the precontemplation (n = 6; 4%) and contemplation (n = 3; 3%) stages.

### Multivariable predictors of smoking behavior

As a group, the collection of covariables in Step 1significantly improved the prediction of smoking status change over the null model (Nagelkerke R^2^ = 0.10; χ² _10_ = 28.25, p < 0.01) (Table 2). The associations between individual covariables and the outcome were substantively similar to the bivariable analyses.

**Table 2:** Odds Ratios Predicting Smoking Status Change

	**Overall Likelihood Ratio Test Chi Square**^**1**^	**Odds Ratio [95 % CI]:Continuous Smoking (0) versus Quit Attempt and Relapse (1)**	**Odds Ratio [95 % CI]: Continuous Smoking (0) versus Successful Change (1)**
**Step 1: Covariables Alone** – Nagelkerke R^2^ = 0.10 (this step stayed the same for all 5 models)			
Age	0.52	1.00 [0.98–1.02]	1.01 [0.98–1.05]
Sex (Male/Female)	6.69*	1.97* [1.16–3.36]	0.97 [0.40–2.38]
Educational Level (> HS/< HS)	2.10	1.20 [0.65–2.22]	0.55 [0.21–1.43]
Race (Nonwhite/White)	10.26**	0.41** [0.24–0.72]	0.67 [0.25–1.77]
Nicotine Dependence	7.76*	0.96 [0.81–1.13]	0.67** [0.50–0.90]
**Model 1: Importance** – Nagelkerke R^2^ = 0.14			
Age	0.29	1.00 [0.98–1.02]	1.01 [0.98–1.05]
Sex (Male/Female)	7.89*	2.13** [1.24–3.68]	1.04 [0.42–2.58]
Educational Level (> HS/< HS)	2.13	1.33 [0.72–2.46]	0.62 [0.24–1.64]
Race (Nonwhite/White)	7.46*	0.46** [0.26–0.81]	0.76 [0.28–2.02]
Nicotine Dependence	6.58^*^	0.99 [0.83–1.17]	0.69* [0.51–0.93]
**Importance**	12.22**	1.17** [1.06–1.29]	1.18† [0.98–1.43]
**Model 2: Readiness** – Nagelkerke R^2^ = 0.13			
Age	0.26	1.00 [0.85–1.20]	1.01 [0.97–1.04]
Sex (Male/Female)	7.38*	2.07** [1.20–3.56]	0.99 [0.40–2.44]
Educational Level (> HS/< HS)	2.47	1.37 [0.73–2.55]	0.61 [0.23–1.59]
Race (Nonwhite/White)	8.64*	0.44** [0.25–0.77]	0.69 [0.26–1.83]
Nicotine Dependence	6.33^*^	1.01 [0.85–1.20]	0.69* [0.51–0.94]
**Readiness**	9.23*	1.13** [1.04–1.23]	1.09 [0.94–1.27]
**Model 3: Confidence** – Nagelkerke R^2^ = 0.13			
Age	0.05	1.00 [0.98–1.02]	1.00 [0.97–1.04]
Sex (Male/Female)	7.37*	2.06** [1.20–3.54]	1.01 [0.41–2.51]
Educational Level (> HS/< HS)	2.16	1.28 [0.69–2.38]	0.59 [0.22–1.55]
Race (Nonwhite/White)	7.79*	0.46** [0.26–0.80]	0.79 [0.30–2.13]
Nicotine Dependence	4.59	1.01 [0.85–1.20]	0.73* [0.54–0.99]
**Confidence**	10.78**	1.10* [1.02–1.19]	1.21* [1.04–1.39]
**Model 4: Motivation Rulers Combined** – Nagelkerke R^2^ = 0.16			
Age	0.20	1.00 [0.98–1.02]	1.01 [0.97–1.04]
Sex (Male/Female)	7.94*	2.15** [1.24–3.72]	1.07 [0.43–2.71]
Educational Level (> HS/< HS)	2.73	1.37 [0.74–2.55]	0.57 [0.21–1.53]
Race (Nonwhite/White)	6.98*	0.47* [0.27–0.83]	0.84 [0.31–2.28]
Nicotine Dependence	4.88^†^	1.01 [0.85–1.20]	0.72* [0.53–0.98]
**Importance**	4.22	1.11^†^ [0.98–1.26]	1.16 [0.93–1.44]
**Readiness**	2.21	1.04 [0.92–1.19]	0.87 [0.70–1.09]
**Confidence**	5.48^†^	1.03 [0.93–1.15]	1.24^*^ [1.03–1.53]
**Model 5: Stage of change** – Nagelkerke R^2^ = 0.20			
Age	0.00	1.00 [0.98–1.02]	1.00 [0.96–1.04]
Sex (Male/Female)	4.72^†^	1.81* [1.04–3.15]	0.99 [0.37–2.62]
Educational Level (> HS/< HS)	2.84	1.26 [0.66–2.35]	0.48 [0.17–1.34]
Race (Nonwhite/White)	8.87^*^	0.43** [0.24–0.76]	0.88 [0.32–2.47]
Nicotine Dependence	5.40^†^	1.01 [0.85–1.20]	0.69* [0.50–0.96]
Contemplation^2^	10.43**	2.50** [1.36–4.59]	0.46 [0.09–2.41]
Preparation^2^	18.81***	3.06** [1.52–6.15]	6.49** [2.20–19.12]

*p < 0.05, **p < 0.01, ^†^ p < 0.10. ^1^All of the likelihood ratio tests performed had two degrees of freedom. ^2^Stage of change was dummy coded, with precontemplation as the reference category.

For each of the ruler models (individual rulers alone and combined), inclusion of the ruler(s) in Step 2 significantly improved prediction of smoking status change (see Table 2, Figure 2). In model 5 (Stage of Change), the inclusion of stage of change in Step 2 also significantly improved prediction of smoking behavior change (Δχ² _4_ = 30.27, p < 0.001). Specifically, contemplaters were significantly more likely to have tried to change but relapse than to have remained a continuous smoker. Preparers were significantly more likely to have tried to change but to have relapsed, or to have successfully changed, than to have remained a continuous smoker.

Supplemental analyses contrasting the two subgroups of changers (i.e., those who tried to quit but relapsed versus successful changers) revealed that, in both models 4 (combined rulers) and 5 (Stage of Change), the only significant predictor of successful quitting over relapsing was nicotine dependence, with more dependent individuals being more likely to relapse (nicotine dependence: OR _motivation rulers_ = 1.40, p < 0.05; OR _stage of change_ = 1.45, p < 0.05).

Comparisons of the Nagelkerke pseudo R^2^ from the four motivation-ruler models (individual rulers and the combined model) and the Stage of Change model revealed that all models provided roughly equivalent fit (all *Z* approximations ≤ 1.41; p values, > 0.05).

## Discussion

Surprisingly, considering the popularity of motivation rulers in clinical practice and their cornerstone in MI-based interventions [1,8], there are very few published studies on their psychometric properties. Our results provide general support for the validity of the three rulers, with the confidence ruler showing slightly better overall performance in predicting behavior change after adjusting for other potential confounding variables.

Construct validity of the rulers was demonstrated through the expected positive associations with stage of change at baseline, which reinforce similar patterns observed in adolescent smokers undergoing addiction treatment [14]. Subjects in the preparation stage of change reported the highest importance, readiness, and confidence. In addition, predictive validity was supported by significant prediction of changes in smoking behavior in the two weeks after the index ED visit. The ability of the motivation rulers, independently and as a group, to predict smoking persisted in the multivariable analysis even after controlling for demographic variables and nicotine dependence. Moreover, the magnitude of this predictive ability was on par with stage of change in the fully adjusted models. This is a robust test of predictive validity, considering that both nicotine dependence and stage of change have historically been strong replicable predictors of smoking behavior [18-20]. In the final combined-ruler model, the confidence ruler appeared to have the strongest and most consistent relation with smoking behavior change. This supports the extant literature showing that self-efficacy, or confidence in one’s ability to change, is a preeminent predictor of change [21,22].

Notably, those who attempted to quit but were unsuccessful and relapsed back to smoking looked remarkably similar in terms of motivation to those who had achieved seven-day abstinence. Both of these groups of changers perceived the importance of smoking cessation and their readiness to quit as significantly higher than those who continued to smoke, but relapsers were not markedly different from successful quitters across these two rulers (e.g., importance and readiness). A similar but slightly more complex trend was noted with confidence: although successful quitters endorsed stronger confidence in quitting than relapsers in the bivariable analysis, thus differentiating the two groups of changers, this effect was attenuated in the multivariable analyses. Only nicotine dependence remained an independent predictor, able to differentiate between those quitters who relapsed back to smoking and those that achieved successful change. This same pattern held true for stage of change. Stage was able to distinguish continuous smokers from both groups of changers but did not differentiate relapsers from successful changers.

As a whole, these data suggest measures of motivation are much better at predicting who will initiate change than they are at predicting transition to successful change. This pattern is consistent with the health-behavior change literature in general, which has prompted recent calls for reformulating traditional health-behavior theories to more proactively distinguish between predictors of behavioral initiation from predictors of behavioral maintenance [23-25]. This same recommendation has been echoed for conceptual model building to study health-behavior change in acute medical settings [26]. Our results suggest that motivational readiness may predict who tries to change, but nicotine dependence predicts who will relapse back to smoking.

In our exploratory analyses, we found that greater nicotine dependence was associated with lower importance, readiness, and confidence ratings—a pattern already observed in the literature [13,14]. It is difficult to know exactly why this association exists. However, some theorists have appealed to cognitive dissonance theory to explain it [27]. Individuals who smoke heavily and are, therefore, strongly dependent on nicotine, and who rate their motivation to change as high, are in a dissonant state. Their behavior, heavy smoking, conflicts with their perceptions, that cessation is important. It is difficult to maintain such a dissonant state for long; one or the other must change. Either motivation wins out, and the individual reduces his or her smoking and, therefore, becomes less dependent, or the individual continues to smoke at a high rate and devalues the importance of cessation. The collective effect is to produce a negative correlation between dependence and motivation. The only way to truly test the dissonance reduction hypotheses is through longitudinal or experimental study designs.

### Limitations

The data were collected in the ED setting, and, consequently, the results should be generalized to other settings with caution. Additional work replicating our results across other settings is needed. The sample sizes for the relapsers and successful quitters were small, making it difficult to detect differences between the predictors, like the motivation rulers. This may have obscured actual differences (i.e., Type II error). Further, the rulers we used were anchored by “0” and “10.” This differs from rulers sometimes employed, which can be anchored by “1.” This difference is subtle and seems unlikely to exert a powerful influence on results or interpretations. Nevertheless, further inquiry into how different rulers perform may be warranted. We used the transtheoretical model’s stages of change to establish construct validity. Although widely used and studied more than any other motivation measure, it is nevertheless controversial, with some scholars suggesting the construct is invalid [28]. Interestingly, one of the main arguments against the stages of change is that motivation is likely to be on a continuum rather than threshold- or stage-based. Rulers and scales that are measured in a more continuous manner are often appealed to as a means of addressing this very limitation. Finally, the effect sizes of some of the association, like the correlations between the readiness rulers and nicotine dependence, were small, prompting caution when interpreting the strength of the results. This limitation is partially mitigated by the fact that, although small, they were generally on par with the effects sizes found in the extant literature on predictors of cessation [19,20,29].

## Conclusion

The study provides support for the reliability and validity of importance, readiness, and confidence rulers in an acute medical setting. As a group, the rulers performed as well as stage of change in predicting smoking behavior change. However, none of the motivation variables differentiated between those who attempted to quit but relapsed from those who were successful quitters. Further research building dynamic models that predict both initiation and maintenance are needed.

## Competing interests

The project described was supported by grant #R21DA020771 from the National Institute on Drug Abuse (NIDA). The content is solely the responsibility of the authors and does not necessarily represent the official views of NIDA or the National Institutes of Health. SLB has served as an expert witness for plaintiffs in litigation against the tobacco companies. None of the other authors have financial conflicts of interest to disclose.

## Authors’ contributions

EDB, SB, AS, and CAC were responsible for study design, execution, and write-up. BA was responsible for data analyses. AG was responsible for assisting in data interpretation and write up. All authors read and approved the final manuscript.
